# How Amino Acids Intercalate in CaFe Layered Double Hydroxides: A Combined RIXS and NEXAFS Study

**DOI:** 10.1002/cphc.202400745

**Published:** 2025-01-28

**Authors:** R. Büchner, A. Born, K. Ruotsalainen, R. Decker, A. Pietzsch

**Affiliations:** ^1^ Institute Methods and Instrumentation for Synchrotron Radiation Research Helmholtz Center Berlin for Materials and Energy Albert-Einstein-Strasse 15 12489 Berlin Germany

**Keywords:** LDH, electronic structure, amino acids, NEXAFS, RIXS

## Abstract

Two‐dimensional layered double hydroxides (LDHs) are ideal candidates for a large number of (bio)catalytic applications due to their flexible composition and easy to tailor properties. Functionality can be achieved by intercalation of amino acids (as the basic units of peptides and proteins). To gain insight on the functionality, we apply resonant inelastic soft x‐ray scattering and near edge x‐ray absorption fine structure spectroscopy to CaFe LDH in its pristine form as well as intercalated with the amino acids proline and cysteine to probe the electronic structure and its changes upon intercalation. We observe the activation of pristine LDH defect states by soft x‐rays and their passivation by the intercalated molecules. The nitrogen at the amino amino is found to form C=NH^+^ bonds and thus generating positive charge at the amino group, moving it away from the positively charged LDH layers. The carboxyl group in cysteine is deprotonated and thus in zwitterionic state after intercalation. This negative charge is used to compensate the positive layer charge. For intercalated proline the spectral signature of a protonated carboxyl group is observed, however, we find orbital overlap to defects at the layer surfaces indicating strong interaction with the carboxyl groups.

## Graphical abstract

Intercalation of amino acids in layered double hydroxides offers a road to functional (bio)catalysts. With resonant soft x‐ray spectroscopy we probe the electronic structure of these systems and observe activation and passivation of electronic states as well as bond formation between the amino acid and the LDH upon intercalation.

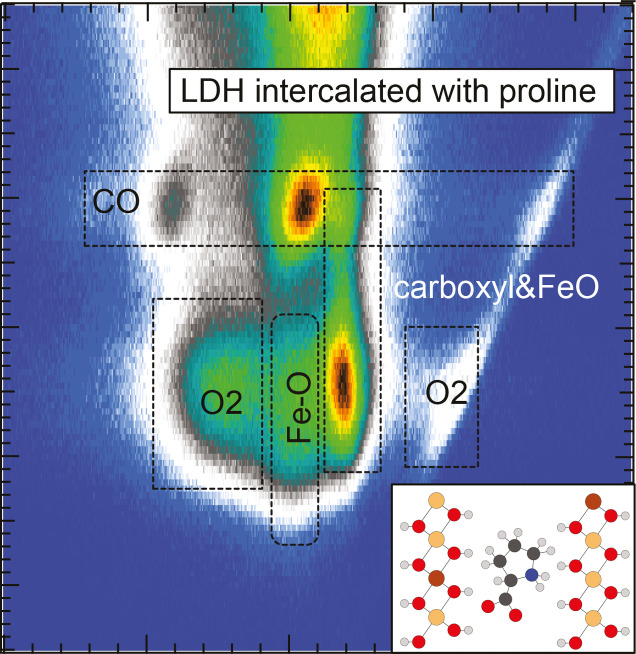

## Introduction

Layered intercalation compounds such as graphite or transition metal dichalcogenites have been explored since the early years of intercalation chemistry.^[1]^ The two‐dimensional layered double hydroxides (LDHs) are anionic plate‐like nanomaterials consisting of di‐ and trivalent metal hydroxides. The synthetic origin and flexible composition of LDHs makes them an obvious choice for materials with controllable (and thus reproducible) tailored properties.^[2]^

LDHs have firstly been explored with the aim of developing new durable catalysts^[3]^ but are now also favored for biomedical applications, such as biosensing,^[4]^ drug carriers and chemotherapeutic agents,^[5]^ or bioimaging and UV protection.^[6]^ For these applications, amino acids are of great interest, being the basic units of peptides and proteins and serving as neurotransmitters in living organisms. Their residues are catalytically active sites for natural enzymes. Apart from that, amino acids have a comparable structure to a variety of organic molecules and are thus appropriate example molecules. L‐proline and L‐cysteine in particular show complementary structural properties and act as a molecular reactor^[7]^ and organocatalyst,^[8]^ respectively.

For intercalation in LDHs it is generally accepted that counter ions are intercalated between the charged layers by electrostatic interaction. However, a study on the encapsulation of clay material by silane revealed that the applied organic coating is covalently linked to the hydroxyl groups of the clay.^[9]^ Therefore it is important to understand how exactly exactly molecules intercalate in layered structures: For catalytically active systems the focus lies on point defects such as oxygen or metal vacancies that are generated during intercalation in the LDH and that can improve or hinder catalytic activity.^[2]^

Soft x‐ray spectroscopy is an ideal tool for investigating these intercalation systems due to its element specificity and chemical selectivity. This allows to disentangle information from different oxygen sites in the LDH and amino acids and thereby obtain information on the local valence electronic structure. The combination of near edge x‐ray absorption fine structure (NEXAFS) and resonant inelastic x‐ray scattering (RIXS) spectroscopy gives a complete map of the unoccupied and occupied valence electronic structure close to the Fermi level and its change upon intercalation.

By means of charting the valence electronic structure of the system locally at different oxygen and nitrogen centers we can gain information on the local chemical environment around these centers and by that draw conclusions on the geometric orientation and charge aggregation of the guest molecules. We can further identify potential docking points for guests by comparing pristine and loaded LDH to see which valence states are occupied upon loading.

In this work, we provide a more fundamental understanding of intercalation of the aminoacids proline and cysteine into the exemplary CaFe‐LDHs. We use soft x‐ray absorption and emission spectroscopy to determine the electronic structure and chemical state of the different oxygen and nitrogen atoms in the LDH layers as well as the ones of the intercalated amino acids which allows us to draw conclusions on the local bonding arrangements in these complex systems. We do not aim to give a complete picture of the intercalated system, but rather show what aspects and questions can be addressed when employing soft x‐ray spectroscopic techniques and how these complement results from other measurement techniques. Despite the limitations of radiation damage, we can still obtain information on the complex LDH and aminoacid/LDH systems that will help to understand how the intercalated molecules interact with the host material.

## Results and Discussion

**Figure 1:**
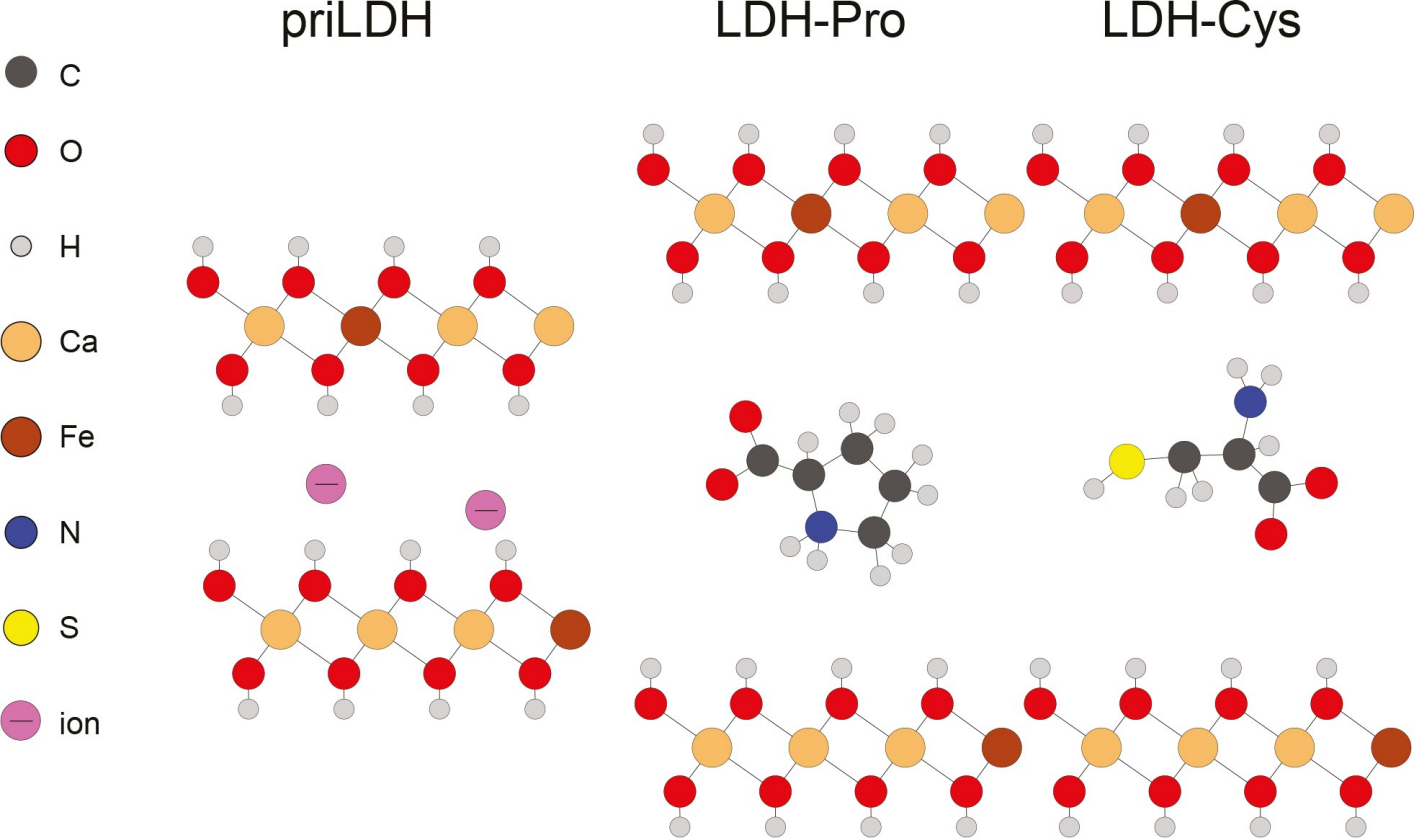
Schematical structure of pristine LDH (left) and LDH with intercalated aminoacids proline (LDH‐pro, center) and cysteine (LDH‐cys, right).

A schematical structure of the LDHs is shown in Figure 1. The pristine LDH (priLDH) compensates the layer charge with negative ions. After intercalation with proline or cysteine, the layer charge in the resulting compounds (LDH‐pro and LDH‐cys, respectively) is compensated by the amio acids.

While the LDH contains many oxygen atoms, most of them are bound to Ca or Fe in the layers. Due to excitation of a core electron, NEXAFS and RIXS are element specific and allow to probe at the different absorption edges with particular sensitivity to the low energy defect states with dangling bonds that are shifted to lower energies due to different screening. In the spectra these show up at the onset of the absorption resonance and are naturally sensitive to intercalation making them ideal for observation of surface and interface alterations.

**Figure 2:**
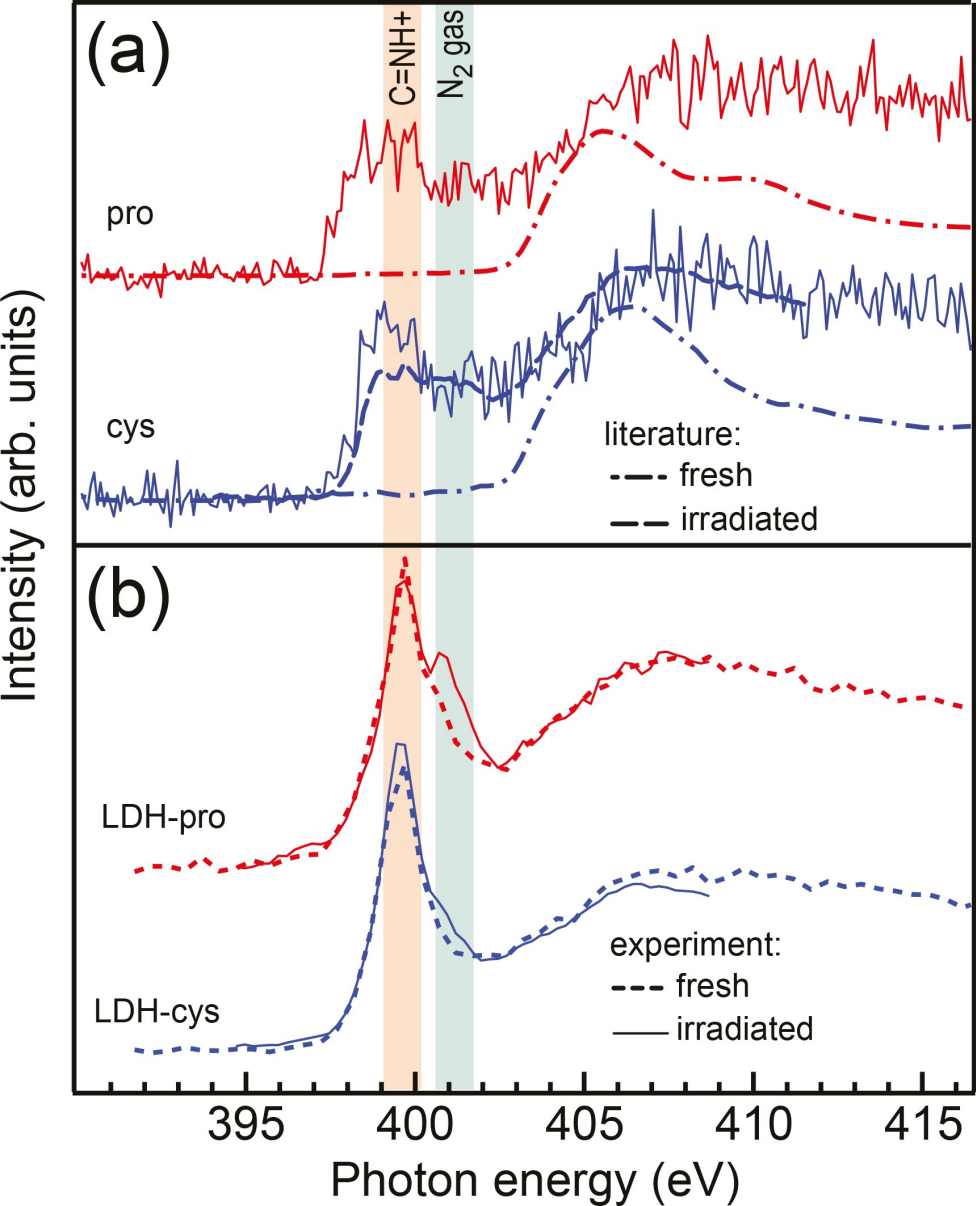
(a) N K‐edge NEXFAS spectra of solid proline and cysteine (solid lines) compared to literature spectra from the pristine amino acids^[10]^ (dash‐dotted lines) and radiation damaged cysteine from Ref. [11] (dashed line). We find that during the measurement time of 25 minutes, the amino acid spectra already show radiation damage. (b)  N K‐edge NEXAFS spectra of LDH‐pro and LDH‐cys for fresh and irradiated samples. Here, a distinct peak at 399.5 eV is observed that is attributed to formation of C=NH^+^ at the amino group due to intercalation. The shoulder at 401 eV is due to formation of quasimolecular N_2_ gas.

In Figure 2 we present N K‐edge NEXAFS spectra of the solid amino acids proline and cysteine as well as the intercalated samples LDH‐pro and LDH‐cys. The nitrogen is for both amino acids only located in the amino group. In panel (a) we compare our amino acid spectra to literature spectra of pristine amino acids^[10]^ with the typical amino group resonance at 406 eV that corresponds to the σ* N−C bonds. When comparing to a literature spectrum of irradiated cysteine^[11]^ we find that on the timescale of our measurements (som 10 minutes), the recorded amino acid spectra already show radiation damage in the form of a broad peak coming up in the range of 398 eV to 402 eV due to the loss of hydrogen atoms and formation of various C−N double and triple bonds.

In our NEXAFS spectra of intercalated cysteine and proline (see Figure 2), we find a strong peak at 399.5 eV which is attributed to the formation of C=NH^+^ bonds at the amino groups^[12]^ and a more or less pronounced shoulder at 401 eV which arises due to the formation of N_2_ gas, see also the SI. The spectral shape differs from that of radiation damaged cysteine.

The C=NH^+^ feature is already present in the fresh intercalated samples (see SI) and is therefore assigned to arise due to intercalation. Due to the long measurement time we are not sensitive to timescales of a few minutes; but on the timescale of some 20 minutes, the intercalated samples appear stable. These positively charged C=NH^+^ groups will orient way from the positively charged layers in the LDH. On the timescale of a few hours the temporal evolution of the spectra is shown in the SI. We observe that while the intensity decreases and the N_2_ signal grows, the overall shape of the NEXAFS spectra is preserved.

**Figure 3:**
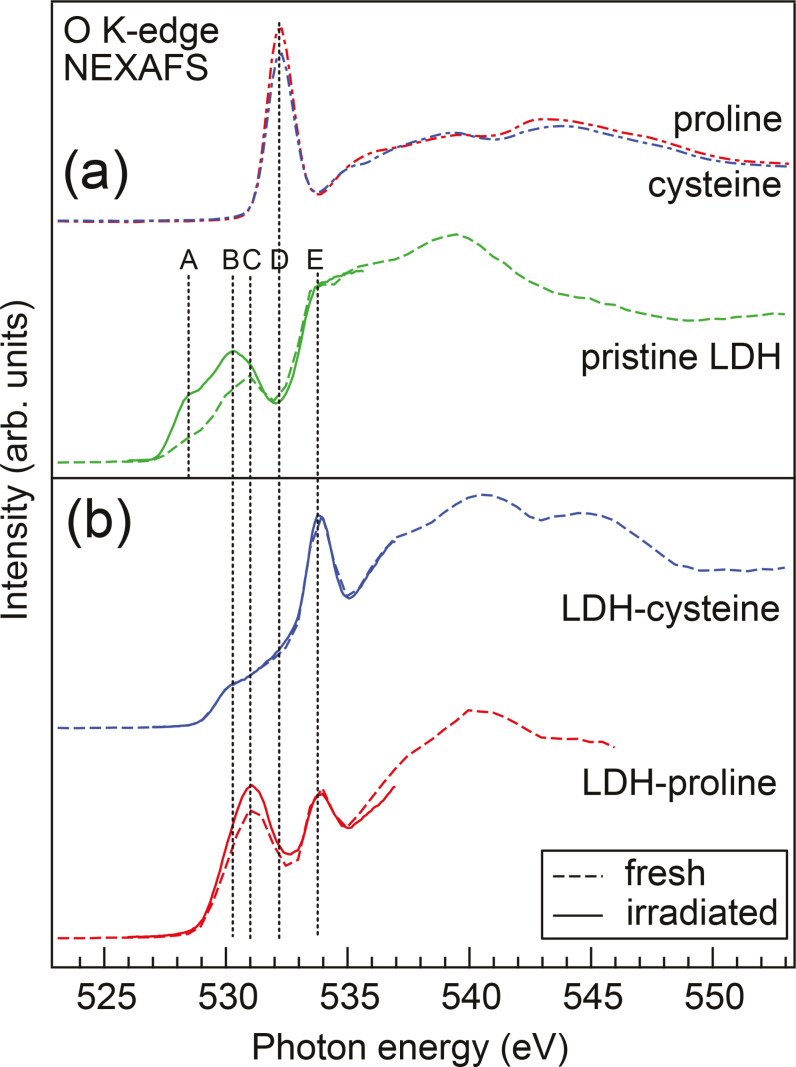
O K‐edge NEXAFS spectra of (a) pristine solid cysteine and proline from Ref. [10] as well as pristine LDH and (b) LDH intercalated with proline or cysteine from fast scans on the fresh samples (dashed lines) and after 1 hour of x‐ray irradiation (solid lines). The absorption features close to the edge are labeled A–E.

The O K‐edge NEXAFS spectra are plotted in Figure 3. Here, panel (a) shows spectra of solid proline and cysteine from Ref. [10] as well as spectra of priLDH. In panel (b) the NEXAFS spectra of the intercalated LDH samples are presented. The absorption features close to the edge are labelled A–E. We will go through these features and use their absorption energy and RIXS spectrum to identify the contributing active groups. The dashed spectra are fast overview measurements and thus mostly undisturbed by radiation damage. After 1 hour of x‐ray irradiation however, signs of x‐ray induced effects are visible (solid lines).

In the spectra of pristine LDH the effects of irradiation induced changes are visible in the energy region 527–532 eV (features A and B in Figure 3): The spectral contribution here is dominated by O−Fe bands of primarily Fe 3d character that are split into $t_{2g}$ and $e_{g}$ orbitals. Depending on the charge state of the Fe atom, the intensity ratio between $t_{2g}$ and $e_{g}$ varies slightly as has been shown for iron oxides.^[13]^

Comparing the fresh and irradiated spectra of pristine LDH (green dashed and solid lines in Figure 3a), we find that the features A and B increase in intensity after 1 hour of irradiation at the O K‐edge. A similar intensity increase has been observed for NiFe and CoFe LDHs during oxygen evolution reaction.^[14]^ There, the authors attribute the spectral increase to a structural change of the LDH layers upon activation where the driving force there is the flexible electronic structure of the surface Fe sites that in synergy with nearest‐neighbor metal sites form O‐bridged Fe‐metal centers and thus increase catalytic activity.

Here, our observations lead to the conclusion that these active Fe−O‐metal sites can also be activated by resonant x‐ray excitation at the oxygen resonance in CaFe LDH. Furthermore, when comparing these results on pristine LDH to the other two samples, we find that intercalation passivates these active defect sites; feature A is missing for both LDH‐pro and LDH‐cys and feature B shows much lower intensity compared to priLDH. Also, the intercalation prohibits the surface activation of the LDH, since the large intensity increase in the Fe−O region after irradiation is missing for LDH‐pro and LDH‐cys. This indicates that these Fe−O sites are the ones involved in the intercalation bonding and are thus passivated by the amino acids.

**Figure 4:**
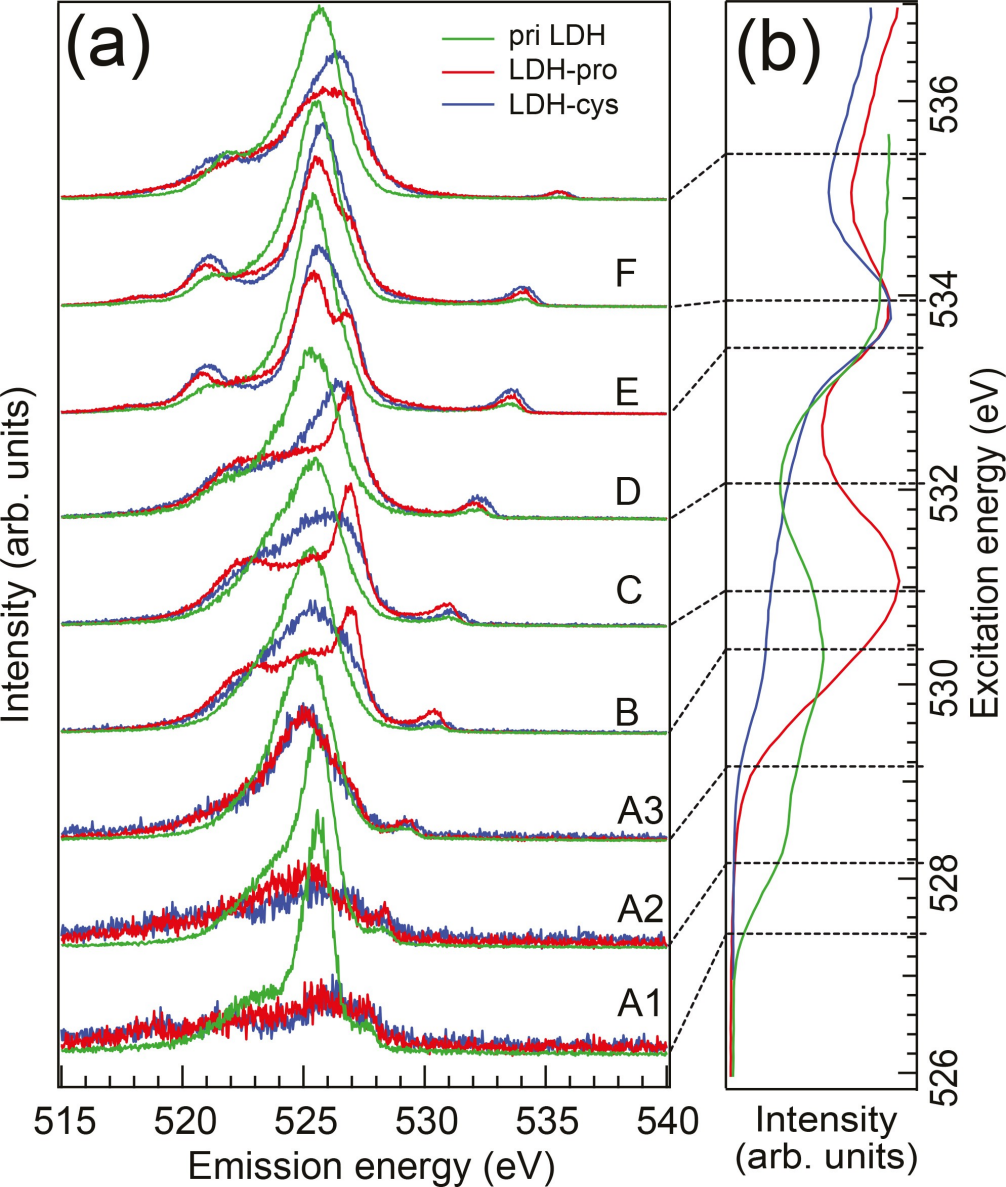
(a) O K‐edge RIXS spectra of priLDH, LDH‐pro and LDH‐cys. The spectra are normalized to their respective area and the absorption features close to the edge are labeled A–F. The excitation energies are marked in the NEXAFS spectra shown in panel (b).

The corresponding RIXS spectra (see Figure 4(a)) show typical iron oxide features for excitation of priLDH at absorption features A1 to A3, see also Ref. [13]. For a direct comparison, see the SI. We find that the RIXS peak becomes narrower for excitation on the onset of the absorption peak. This means that for highly detuned excitation we probe a subset of localized Fe−O orbitals, probably connected to defect states. These are the orbitals that are involved in the intercalation bond to the amino acids: the overlap of the O2p to the Fe3d is reduced significantly upon intercalation, leading for them to disappear from the oxygen NEXAFS and RIXS spectra. This indicates a structural change in the LDH after intercalation.

Absorption feature C is most prominent in LDH‐pro; whereas the LDH‐cys and priLDH only show a small shoulder at this energy. This feature is attributed to radiation‐induced production of quasimolecular oxygen as has been observed for e. g. amorphous metal oxides.^[15]^ There, pairs of oxygen atoms are formed that act as hole traps, creating localized states at the top and bottom of the valence band. Its intensity increases upon irradiation only for LDH‐pro. For priLDH and LDH‐cys these states seem to be protected from radiation‐induced change. In battery materials, quasimolecular oxygen signal has also been found during cycling^[16]^ where the oxygen pairs are formed for charge compensation of intercalated ions. In our case, the oxygen pairs are potentially used to compensate charge in LDH‐pro as well as will be discussed later.

The RIXS spectra excited at absorption feature C (see Figure 4(a)) show apart from the Fe−O signal also a contribution from quasimolecular oxygen in the energy region 521–524 eV. A comparison with RIXS of molecular O_2_ is shown in the SI. The strong feature at 527 eV in the same RIXS spectra is due to the amino acid carboxyl group that has its resonance energy at 532.2 eV (feature D). Figure 3(a) shows the NEXAFS spectra of solid cysteine and proline from Ref. [10]. Those are dominated by the resonance from the carboxylate group, which is in its zwitterionic COO− state for solid amino acids. The resonance shifts 0.3 eV to lower energies for a protonated carboxyl (COOH) group.^[17]^

Radiation damage is always an issue when investigating organic materials with x‐rays. A radiation damage study on pure solid cysteine^[11]^ shows that NEXAFS spectra at the O K‐edge preserve their general shape under irradiation but lose intensity (90 % within 20 minutes). This indicates that the oxygen is released from the amino acid during irradiation in the form of gaseous decomposition products. The remaining (probed) oxygen is, however, still present in a carboxyl environment. Our measurements on intercalated amino acids only show small spectral variation compared to pure amino acids: an increase of the quasimolecular O_2_ signal for LDH‐pro and no change for LDH‐cys. This indicated that despite the radiation damage, the system reaches an equilibrium where information on the bonding and electronic structure of these compounds can be gained with NEXAFS and RIXS spectroscopy.

Due to overlapping electronic states, we cannot distinguish whether the intercalated amino acids are protonated or deprotonated only from the NEXAFS spectra. However, in the RIXS spectra, both configurations have very distinct fingerprints, as has been shown for cysteine^[18]^ and proline^[19]^ in solution: The RIXS of the protonated carboxyl group shows one oxygen emission peak whereas there is a double peak in the deprotonated case. In our case, we find that the LDH‐pro RIXS shows a main peak at 527 eV whereas the corresponding feature is broader and shifted to lower emission energies for LDH‐cys and absent for priLDH which is dominated by LDH Ca−O signal.

We now want to identify the contributions of the amino acids to the LDH‐pro and LDH‐cys RIXS spectra at feature D as well as gain information on the respective carboxyl group protonation. For acetic acid it has been shown that RIXS spectra of the anionic (high pH) and neutral (low pH) molecules show distinct fingerprint features corresponding to the protonation state of the carboxyl group. Spectra of intermediate pH can be reproduced by summing spectra of the two forms since those do not interact.^[20]^

**Figure 5:**
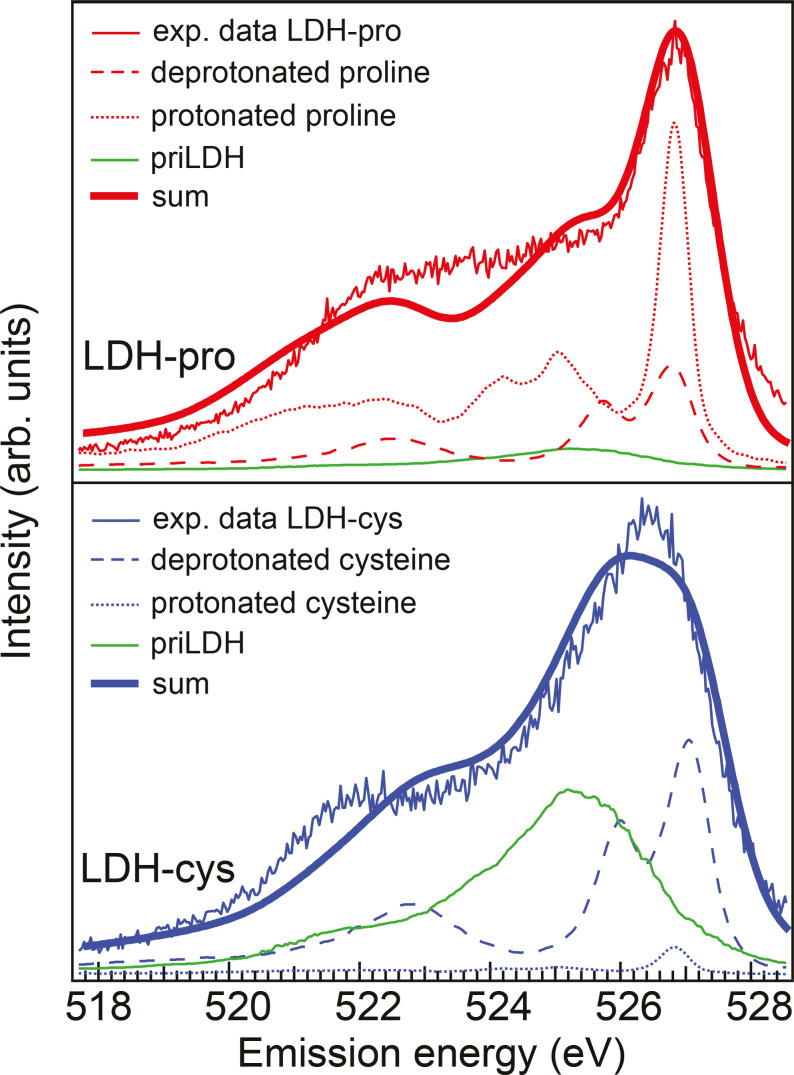
RIXS spectra of LDH‐pro and LDH‐cys excited at the carboxyl group resonance (absorption feature D). The protonated COOH and deprotonated COO^−^ species have different RIXS fingerprints which are used to estimate the respective contribution to the LDH‐cys and LDH‐pro spectra. We fit these with RIXS spectra from protonated and deprotonated cysteine (from Ref. [18]) and proline (from Ref. [19]) together with background from pristine LDH. The resulting sum was convoluted with the instrumental energy resolution of 0.5 eV. We find that in LDH‐pro the signal of the protonated COOH dominates the spectrum while for LDH‐cys the contribution from COO^−^ is highest, albeit with a significant background from pristine LDH, suggesting that the amount of intercalated amino acids is higher for LDH‐pro than for LDH‐cys.

We apply this technique to our results and fit the RIXS spectra of LDH‐pro and LDH‐cys with a sum of the respective protonated and deprotonated amino acid RIXS spectra from literature[^18^, ^19^] together with a possible background contribution from the pristine LDH. The fitted sum was convoluted with the instrumental energy resolution of 0.5 eV and the results are shown in Figure 5; the fitted weight percentages are given in Table 1. We find that the spectrum of LDH‐pro mostly consists of protonated COOH while LDH‐cys shows a much stronger signal from deprotonated COO^−^ over a high LDH background. The differences between the sum of the different spectral contributions and the experimental data are attributed to contributions from other oxygen species with close‐lying absorption resonances, e. g. the tail of CO absorption as well as potential (small) contamination of carbonate. We chose not to include these into the sum for simplicity reasons. However, we can use the spectral fingerprints to get indication on the protonation state of the carboxyl group of the amino acids which helps to understand how the amino acids orient inside the LDH and where Coulomb interaction or other bonding could play a role.

**Table 1:** Spectral weight of protonated and deprotonated amino acids for LDH‐pro and LDH‐cys excited at 532.2 eV (absorption feature D).

	LDH‐pro	LDH‐cys
Protonated	0.69	0.04
Deprotonated	0.21	0.34
Background LDH	0.10	0.62

It has to be pointed out that the oxygen spectral fingerprint of the protonated carboxyl group does not necessarily imply that the carboxyl is protonated, but rather that the two oxygen atoms at the carboxylic group are distinguishable, i. e. not in zwitterionic configuration.

In the RIXS spectra, the emission feature at 527 eV prominent in LDH‐pro (see SI) is associated with the protonated aminoacid COOH group. The absorption energy for oxygen in COOH is 532.3 eV which is in line with the NEXAFS spectra of pristine proline and cysteine. However, we observe the COOH emission feature is already visible starting at about 530 eV photon energy. It almost overlaps with the emission from the quasimolecular O_2_ gas (and partly the Fe−O $e_{g}$ ), but is not part of the O_2_ spectrum, see SI, nor does it originate from the LDH.

The emission feature decreases in intensity before getting stronger again close to the COOH excitation. This means that while there is the possibility of direct excitation of oxygen in the COOH group, there is another excitation pathway involving the quasimolecular oxygen and to some extend the Fe−O. We therefore propose significant overlap of the COOH with the O=O (and partially Fe−O) valence orbitals so that a local core hole at the Fe−O or O=O can be filled by an electron from the COOH group. This is visible in our spectra through the resonating behavior of the COOH emission feature with excitation over the O_2_ and COOH resonances. Our result can thus also point towards a new bond between one oxygen in the carboxyl group in the proline and the LDH. Similar features have been observed for oleic acid coated iron oxide nanoparticles,^[13]^ see also SI for comparison.

**Figure 6:**
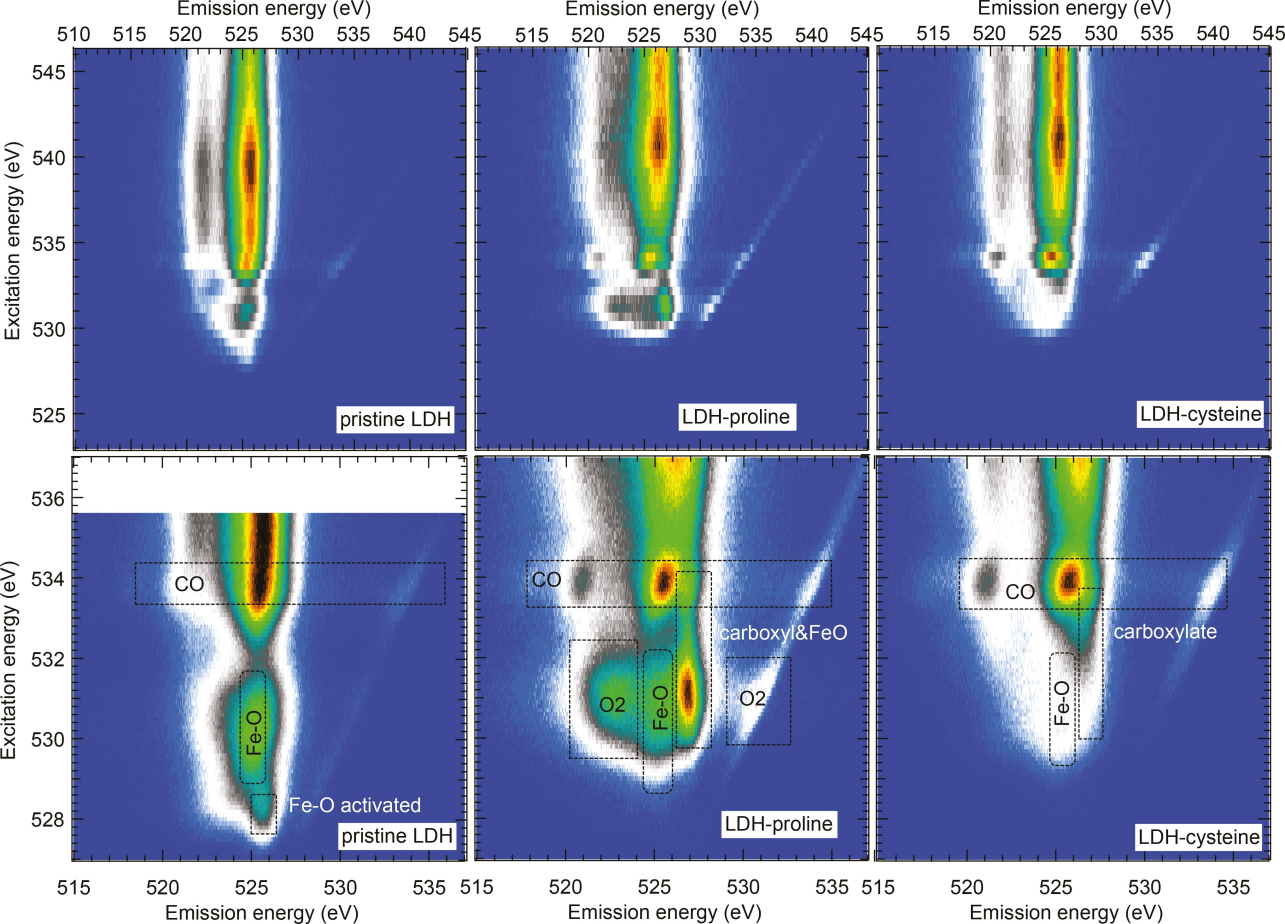
RIXSmaps for priLDH (left), LDH‐pro (center), and LDH‐cys (right). Overview maps are presented in the upper part; the lower part shows detailed zoom‐ins on the near‐edge features with assignments of the most prominent features marked in boxes. The diagonal lines stem from elastic scattering.

To visualize this, we plot the O K‐edge NEXAFS and RIXS in RIXSmaps, see Figure 6. In the lower panels the near edge absorption and emission features are shown in detail together with their assigned contributions. The corresponding absorption energies are given in Table 2. In the case of LDH‐pro we observe the neutral carboxyl COOH RIXS fingerprint peak (527 eV emission energy) at the carboxyl absorption resonance at 532.5 eV excitation energy, but also at 531 eV excitation energy which corresponds to the resonances of quasimolecular O_2_ and Fe−O $e_{g}$ . The potential formation of the bond of the Fe to the proline rises the question how the charges in the LDH are compensated. Here we suggest that the quasimolecular oxygen acting as a hole trap (that shows indeed strongest signal in LDH‐pro) takes the role of charge compensation in this system.

**Table 2:** Assignment of oxygen absorption features in priLDH, LDH‐pro and LDH‐cys.

Feature	Energy (eV)	Assignment
A	528.4	Fe−O $t_{2g}$
B	530.3	Fe−O $e_{g}$
C	531.0	Quasimolecular O_2_
D	532.2	Amino acid carboxyl group
E	533.9	CO gas

The last absorption resonance to be discussed is feature E at 533.9 eV which is associated with the generation of a CO gas‐like spectral shape, in particular for LDH with intercalated amino acids (see SI). The relative intensity of this feature is much stronger for LDH with intercalated amino acids than for the pristine LDH and we assume that part of the amino acids are dissociated during the intercalation and measurement process and the fragments adsorb on the LDH surface as CO. Since adsorbed CO only has a very low heat of adsorption, a gas‐like spectral shape is expected. The relative intensity of feature E does not change when comparing fresh and irradiated samples, so the CO generation does not seem to occur on the timescale of our measurements. It could either be very fast or not related to x‐rays at all. The latter case would prove that intercalation does indeed protect amino acids from radiation damage efficiently.

## Conclusions

NEXAFS and RIXS allow to extract fingerprint features in both absorption and emission to disentangle spectral contributions in complex systems. For the system of pristine Ca−Fe LDH, we have shown the signature of activation of catalytic Fe sites upon irradiation with soft x‐rays and that intercalation passivates these sites and thus prohibits activation. Intercalation of the two simple amnio acids cysteine and proline has revealed different intercalation structures through different spectroscopic fingerprints of the amino acid amino and carboxyl groups. At the amino group, C=NH^+^ bonds are formed upon intercalation that are be stable upon irradiation over several hours. These positively charged groups will orient way from the positively charged layers in the intercalation complex. The oxygen atoms at the carboxyl group are found to be in zwitterionic COO^−^ state for LDH‐cys and are thus here the main actor in compensating the layer charge of the LDH. The spectral fingerprint of the carboxyl group in LDH‐pro is that of a neutral COOH group, meaning layer charge compensation does not include Coulomb interaction with the proline carboxyl group. But instead, in LDH‐pro orbital overlap is found between the oxygen atoms in the carboxyl group and O_2_‐like defects and Fe−O $e_{g}$ . This indicates strong interaction between these partners and a more covalent bond of proline to the LDH is suggested.

## Experimental

The intercalation compounds were provided by I. Palinko from University of Szeged. The Ca_3_Fe‐LDHs were prepared by coprecipitation as described in Ref. [21] with a molar ratio of Ca : Fe of 3 : 1. The aminoacids proline and cysteine were intercalated via the ion‐exchange method. The resulting powder samples were attached to the sample holder with carbon tape.

The soft x‐ray spectroscopic measurements were performed at the oxygen and nitrogen K‐edge in partial fluorescence yield and carried out at beamline U49‐2 PGM‐1 at the synchrotron BESSY II in Berlin, Germany, using the SolidFlexRIXS endstation.^[22]^ The overall energy resolution was 150 meV for NEXAFS and 500 meV for RIXS.

## Conflict of Interests

There is no conflict of interest.

1

## Supporting information

As a service to our authors and readers, this journal provides supporting information supplied by the authors. Such materials are peer reviewed and may be re‐organized for online delivery, but are not copy‐edited or typeset. Technical support issues arising from supporting information (other than missing files) should be addressed to the authors.

Supporting Information

## Data Availability

The data that support the findings of this study are available from the corresponding author upon reasonable request.
