# Supplementary material for: How Amino Acids Intercalate in CaFe Layered Double Hydroxides: A Combined RIXS and NEXAFS Study
Source: Chemphyschem. 2025 Jan 28;26(6):e202400745. doi: 10.1002/cphc.202400745 (PMC11913466; doi:10.1002/cphc.202400745)
Supplement: Supplementary file 1 — Supporting Information [file CPHC-26-e202400745-s001.pdf]

ChemPhysChem

Supporting Information

How Amino Acids Intercalate in CaFe Layered Double Hydroxides: A Combined RIXS and NEXAFS Study

R. Büchner, A. Born, K. Ruotsalainen, R. Decker, and A. Pietzsch*

Supporting Information: How amino acids intercalate in CaFe layered double hydroxides: A combined RIXS and NEXAFS study

Robby Büchner, Artur Born, Kari Ruotsalainen, Regis Decker, and Annette Pietzsch*

Institute Methods and Instrumentation for Synchrotron Radiation Research, Helmholtz Center Berlin for Materials and Energy, Albert-Einstein-Strasse 15, 12489 Berlin, Germany

E-mail: annette.pietzsch@helmhholtz-berlin.de

Supporting Information Available

Photon energy calibration

The total energy scale of our spectra were calibrated in the following way: For the N-edge measurements, we observe in NEXAFS of LDH-cys with high resolution an absorption feature stemming from gas-like N_2 - resulting probably from residuous N_2 in the layers, see Figure-SI S1. This quasimolecular system shows the characteristic absorption shape of N_2 with sharp "fingers" where the first resonance is centered at 400.8 eV. We aligned all spectra to the high resolution LDH-cys spectrum, this gives the absolute photon energy scale. The spectra from literature were all used with their intrinsic energy scale.

For the O-edge, we used CO absorption with high resolution for the absolute energy calibration where the highest vibrational peak of the first resonance is at 534.15 eV ([S1]). O K-edge reference spectra measured on the clay material montmorillonite show a distinct absorption peak at 525.95 eV; the montmorillonite NEXAFS was measured at all beamtimes to be able to align the oxygen spectra. Again, the spectra from literature were used without further shifting in energy. The dispersion energy scale of the RIXS spectra was determined by fitting the elastic line in the RIXSmap with a gaussian and assigning the nominal energy from the absolute photon energy scale.

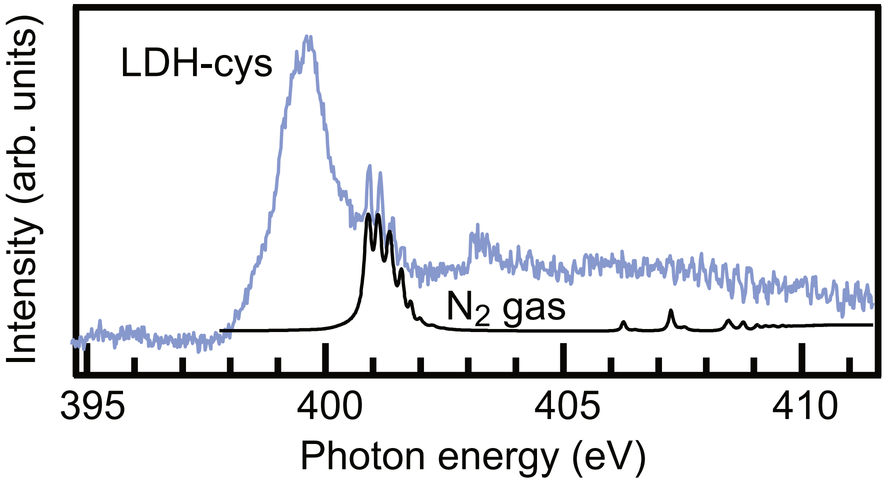

Figure-SI S1: High resolution N-edge NEXAFS spectra of LDH-cys and N_2 gas. We observe the formation of quasimolecular N_2 in LDH-cys upon irradiation where the single vibrational peaks follow those of pure N_2 gas in intensity and energy. The energy of the first N_2 vibrational peak is used as calibration of the total photon energy for all N-edge spectra.

Radiation damage

As organic molecules amino acids are sensitive to radiation damage. A radiation damage study on pure solid cysteine[S3] show that NEXAFS spectra at the O K-edge preserve their general shape under irradiation but lose intensity (90% within 20 minutes). This indicates that the oxygen is released from the amino acid in the form of gaseous decomposition products. The remaining (probed) oxygen is, however, still present in a carboxyl environment,

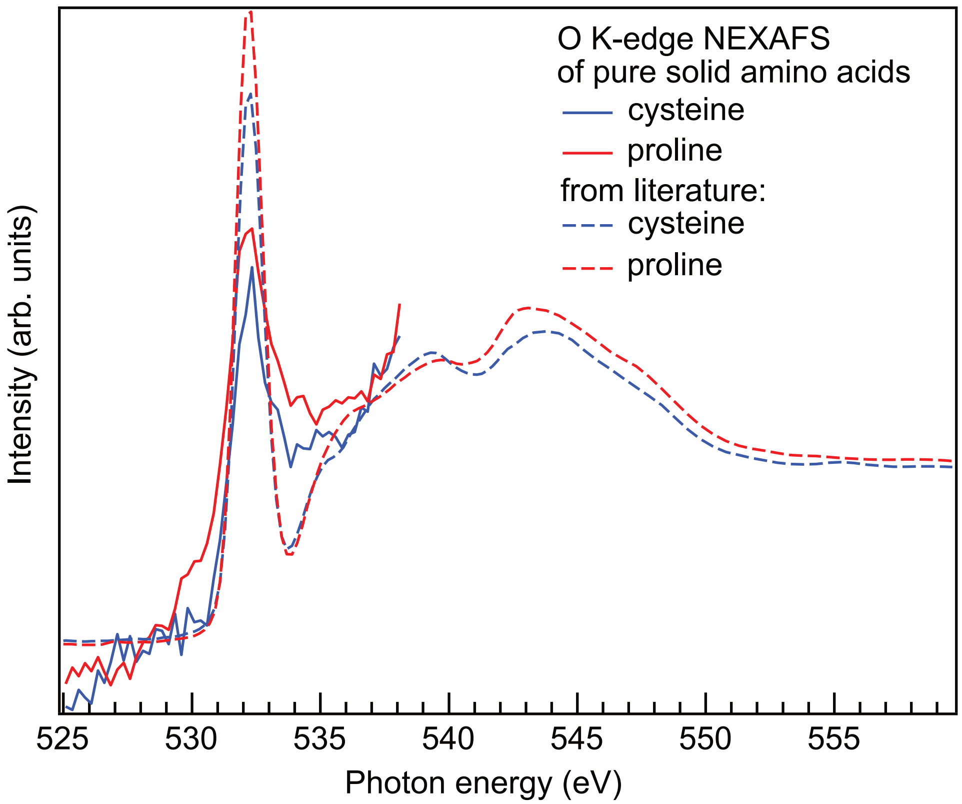

Figure-SI S2: O K-edge NEXFAS spectra of solid proline and cysteine (solid lines) compared to literature spectra from[S2] (dashed lines). We find that while our spectra show radiation damage in the decreased intensity, the overall shape of the spectra and the position of the first (carboxyl) resonance stays the same. This is in line with a previous study on radiation damage in amino acids[S3].

see Figure-SI S2.

NEXAFS spectra at the N-edge of pure amino acids show a series of new peaks in the range of 399-403 eV arising which are attributed to N $1s \rightarrow \pi^*$ transitions of various N-C double and triple bonds. Our N-edge NEXAFS spectra of pure cysteine and proline reflect this fact. Comparison to the literature spectrum of cysteine measured after 14 minutes of irradiation from Ref.[S3], gives a general similar shape.

In the NEXAFS spectra of intercalated cysteine and proline, we find besides the typical amino group resonance at 406 eV that corresponds to the sigma* N-C bonds a strong peak at 399.5 eV which is attributed to the formation of C=NH+ bonds[S4] and a more or less pronounced shoulder at 401 eV which arises due to the formation of N2 gas. The C=NH+

feature at 399.5 eV is already present in the fresh samples, see Figure-SI S3 and is therefore assigned to arise due to intercalation. Due to the long measurement time we are not sensitive to timescales of a few minutes; but on the timescale of some 20 minutes, the intercalated samples appear stable and intercalation seems to induce formation of C=N-H+ bonds at the amino groups.

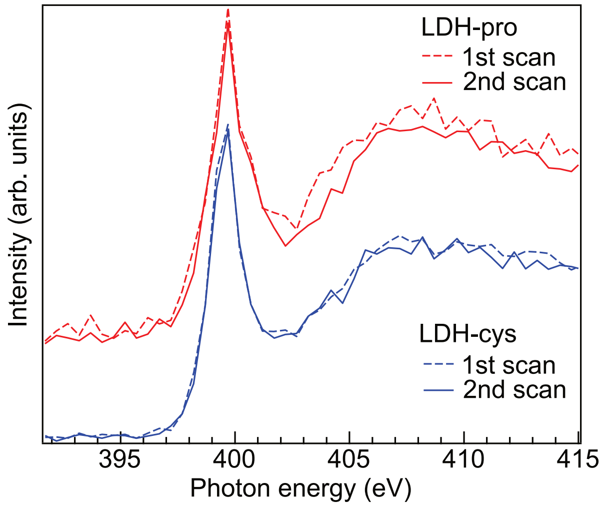

Figure-SI S3: N K-edge NEXFAS spectra of fresh LDH-cys and LDH-pro measured with partial fluorescence yield. The respective consecutive spectra (25 minutes each) show similar spectral shape and no sign of further radiation damage.

The temporal evolution of these spectra are shown in Figure-SI S4. We observe that while the overall intensity decreases and the relative intensity of the N_2 signal increases, the overall shape of the NEXAFS spectra (see Figure-SI S4 (b) and (d)) is preserved. Due to the long measurement time of RIXS we cannot conclude much about timescales of a few minutes, but on the timescale of a few hours, the intercalated samples appear rather stable and intercalation seems to induce formation of C=N-H+ bonds at the amino groups. These positively charged groups will orient way from the positively charged LDH layers while the negatively charged COO^- groups move towards the layers and interact.

NEXAFS at the carbon edge has not been recorded due to low beamline intensity. An additional problem is that carbon contamination from sample handling blurs the real amino acid signal and makes disentanglement of signal contributions extremely challenging. From

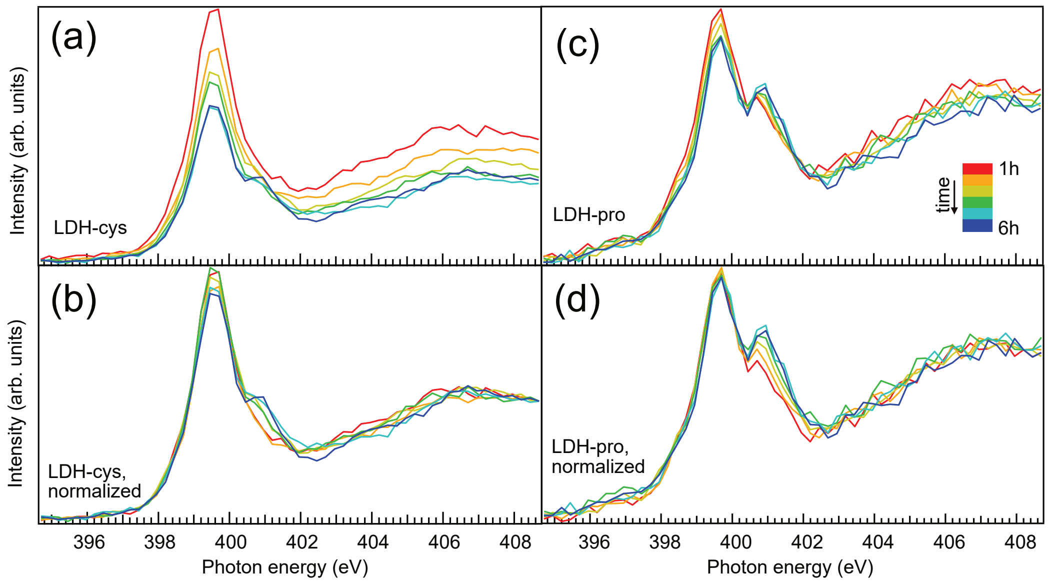

Figure-SI S4: N K-edge NEXFAS spectra of LDH-cys (a and b) and LDH-pro (c and d). The upper two panels a and c show the raw spectra while in the lower two panels b and d the spectra are normalized to the edge jump. LDH-cys shows an intensity decrease of the nitrogen signal in the first 4 hours that then bottoms after the 5h spectrum (light blue line). For LDH-pro, the main resonance at 399.5 eV decreases slightly while the shoulder at 401 eV increases with about the same amount. The normalized spectra show that for both LDH-cys and LDH-pro the spectral shape is preserved apart from a growing shoulder at 401 eV that is attributed to quasimolecular N_2.

literature, we note that for pure amino acids, the carbon is found to loose hydrogen atoms upon irradiation[S3], which is mirrored in our observation of formation of C=N-H+ bonds.

O K-edge RIXS spectra

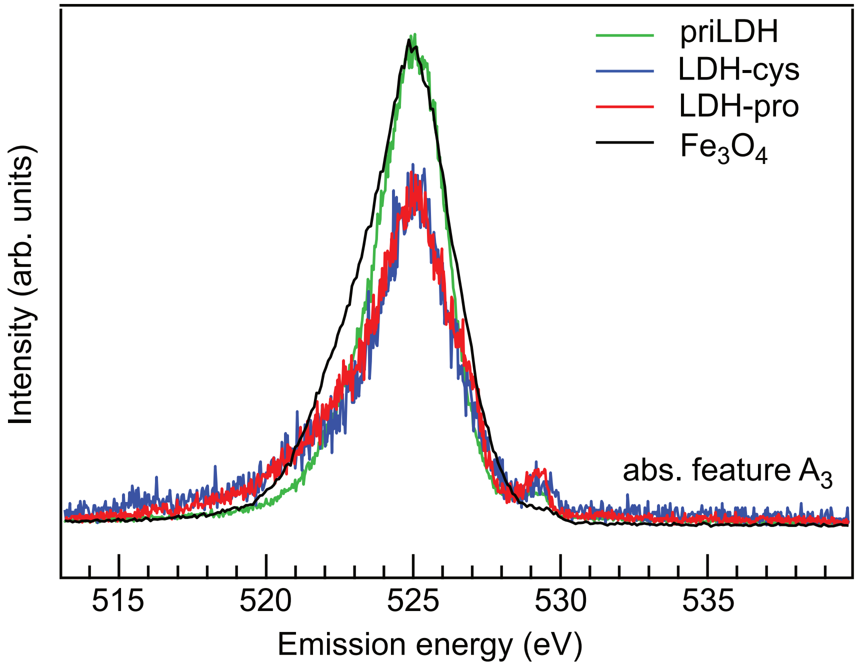

Figure-SI S5: O K-edge RIXS spectra excited at absorption feature A_3 of priLDH, LDH-pro and LDH-cys compared to RIXS spectra of Fe_3O_4 from Ref.[S5]. All spectra show the clear signature of iron oxide.

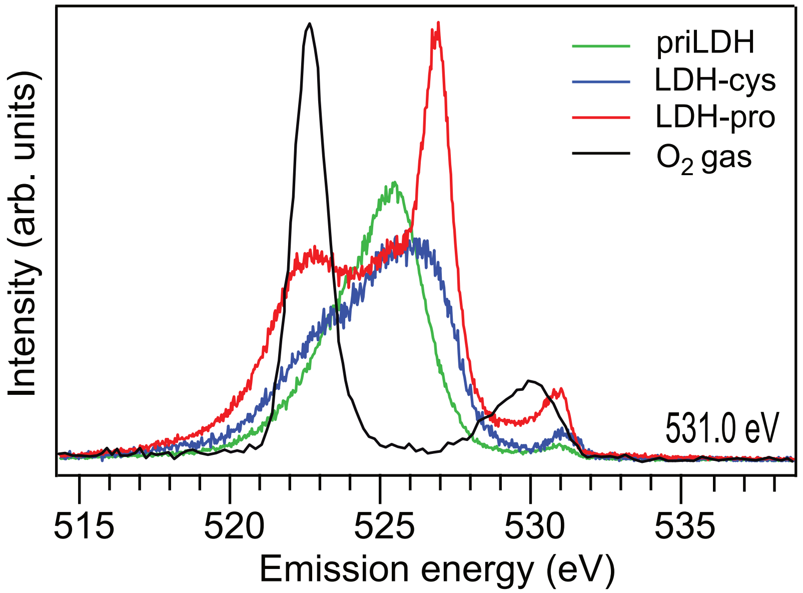

Figure-SI S6: O K-edge RIXS spectra excited at absorption feature C of priLDH, LDH-pro and LDH-cys compared to a resonant RIXS spectrum of O_2 gas from Ref.[S6] excited at absorption feature C. LDH-pro shows an additional signature of gasphase O_2 in the energy region 521-524 eV.

References

(S1) Prince, K.; Richter, R.; de Simone, M.; Alagia, M.; Coreno, M. Near Edge X-ray Absorption Spectra of Some Small Polyatomic Molecules. *J. Phys. Chem. A* **2003**, *2003*, 1955.

(S2) Zubavichus, Y.; Shaporenko, A.; Grunze, M.; Zharnikov, M. Innershell absorption spectroscopy of amino acids at all relevant absorption edges. *Journal of Physical Chemistry A* **2005**, *109*, 6998.

(S3) Zubavichus, Y.; Fuchs, O.; Weinhardt, L.; Heske, C.; Umbach, E.; Denlinger, J. D.; Grunze, M. Soft X-Ray-Induced Decomposition of Amino Acids: An XPS, Mass Spectrometry, and NEXAFS Study. *Radiation Research* **2004**, *161*, 346.

(S4) Graf, N.; Yegen, E.; Gross, T.; Lippitz, A.; Weigel, W.; Krakert, S.; Terfort, A.;

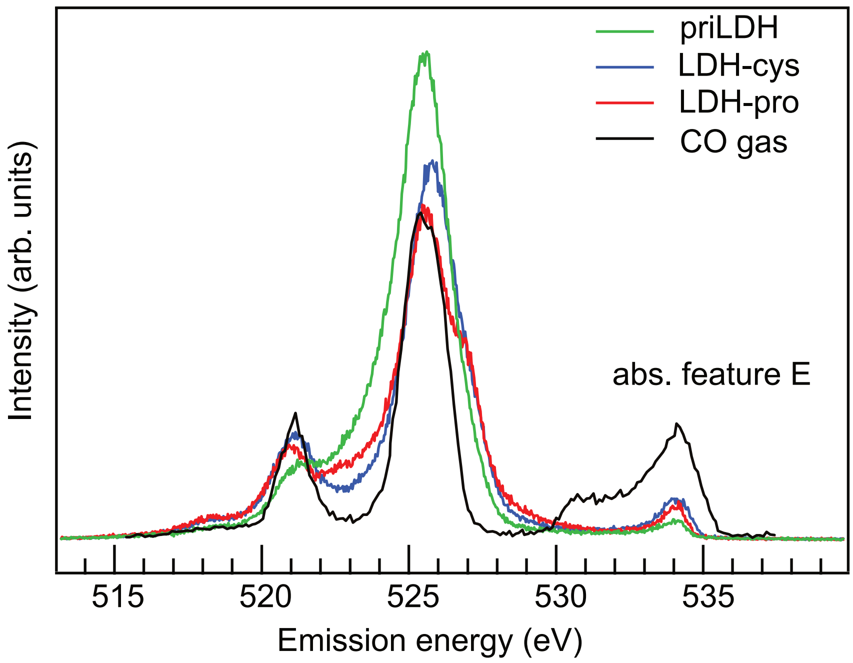

Figure-SI S7: O K-edge RIXS spectra excited at absorption feature C of priLDH, LDH-pro and LDH-cys compared to a resonant RIXS spectrum of O_2 gas from Ref.[S7] excited at absorption feature E. Both the LDH-pro and LDH-cys spectra show close correlation to the CO gasphase spectra together with additional intensity at the elastic peak whereas in the spectrum of priLDH only a slight resemblance to the CO spectrum is detected.

Unger, W. E. S. XPS and NEXAFS studies of aliphatic and aromatic amine species on functionalized surfaces. *Surf. Sci.* **2009**, *603*, 2849.

(S5) Gilbert, B.; Katz, J. E.; Denlinger, J. D.; Yin, Y.; Falcone, R.; Waychunas, G. A. Soft X-ray Spectroscopy Study of the Electronic Structure of Oxidized and Partially Oxidized Magnetite Nanoparticles. *J. Phys. Chem. C* **2010**, *114*, 21994.

(S6) Glans, P.; Gunnelin, K.; Skytt, P.; Guo, J.-H.; Wassdahl, N.; Nordgren, J.; Ågren, H.; Gel'mukhanov, F.; Warwick, T.; Rotenberg, E. Resonant X-Ray Emission Spectroscopy of Molecular Oxygen. *Phys. Rev. Lett.* **1996**, *76*, 2448.

(S7) Skytt, P.; Glans, P.; Gunnelin, K.; Guo, J.; Nordgren, J.; Luo, Y.; Ågren, H. Role of screening and angular distributions in resonant x-ray emission of CO. *Phys. Rev. A* **1997**, *55*, 134.

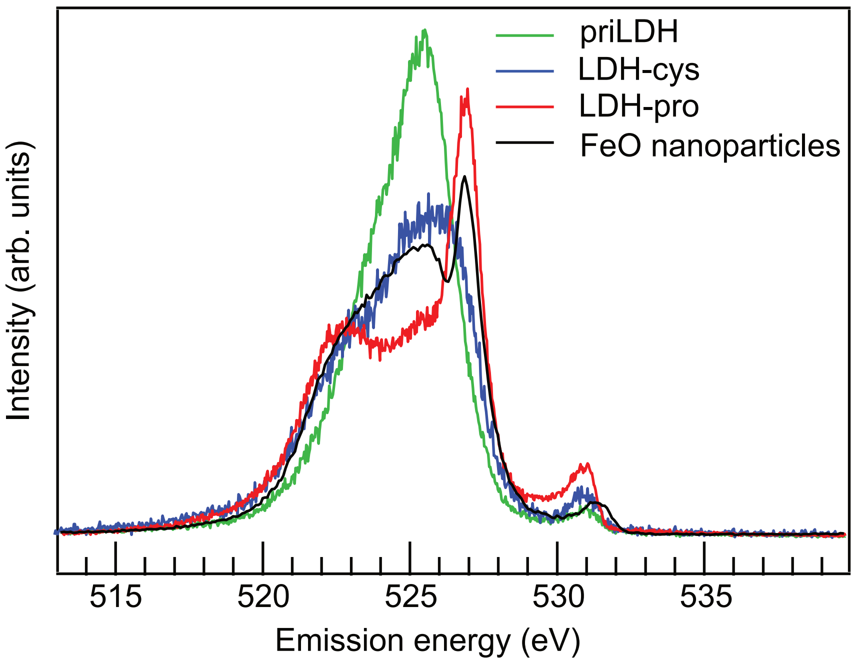

Figure-SI S8: O K-edge RIXS spectra excited at absorption feature D of priLDH, LDH-pro and LDH-cys compared to a resonant RIXS spectrum of iron oxide nanoparticles coated with oleic acid from Ref.[S5] excited at absorption feature D. LDH-pro shows the characteristic peak of the carboxyl group at 527 eV. The elastic line signal of the nanoparticle spectrum is shifted with respect to our data due to the fact that the iron oxide nanoparticles were measured at a slightly different excitation energy.
